# Monthly Record of Deaths in the City of Buffalo

**Published:** 1857-09

**Authors:** C. L. Dayton

**Affiliations:** Health Physician


ART. X.—*Report of Mortality in Buffalo for the month of July*, 1857. By C. L. DAYTON, M. D., Health Physician.

| DISEASES. | No. | Males. | Females. |
|---|---|---|---|
| Accidens, | 7 | 7 | |
| " Asphyxia Immersorum, | 12 | 10 | 2 |
| Croup, | 1 | | 1 |
| Cholera Infantum, | 10 | 5 | 5 |
| Cholera Morbus, | 2 | 2 | |
| Cephalitis, | 4 | 1 | 3 |
| Coup de Soleil, | 2 | 1 | 1 |
| Cancrum Oris, | 1 | | 1 |
| Dysenteria, | 2 | 1 | 1 |
| Dentitio, | 2 | 2 | |
| Diarrhœa, | 6 | 3 | 3 |
| Debilitas Senilis, | 4 | 2 | 2 |
| Eclampsia, | 14 | 7 | 7 |
| Enteritis, | 1 | 1 | |
| Febris Typhoid, | 3 | 2 | 1 |
| " Typhus, | 2 | 1 | 1 |
| " Scarlatina, | 10 | 6 | 4 |
| " Puerperalis, | 1 | | 1 |
| Gastritis, | 1 | | 1 |
| Gangrena Pulmonum, | 1 | | 1 |
| Hydrops, | 2 | 1 | 1 |
| Hydrocephalus, | 3 | 1 | 2 |
| Hyperæmia Cerebralis, | 4 | 3 | 1 |
| Hyperæmia Pulmonalis, | 2 | 2 | |
| Intemperance et Delirium Tremens, | 5 | 4 | 1 |
| Ignotus, | 15 | 4 | 11 |
| Marasmus, | 4 | 1 | 3 |
| Morbus Cordis, | 1 | 1 | |
| Necrosis Femoris, | 1 | 1 | |
| Pertussis, | 1 | | 1 |
| Pneumonitis, | 6 | 1 | 5 |
| Parturitio, | 1 | | 1 |
| Rubeola, | 3 | | 3 |
| Still-born, | 8 | 5 | 1 |

### REGISTER OF MORTALITY—Continued.

| DISEASES. | No. | Males. | Females. |
|---|---|---|---|
| Tuberculosis Pulmonalis, | 26 | 8 | 18 |
| Tumor Abdominis, | 1 | 1 | |
| Tetanus, | 1 | 1 | |
| Total, | 170 | | |

### SEXES.

| | |
|---|---|
| Males, | 85 |
| Females, | 83 |
| Sex not given, | 2 |
| Total, | 170 |

### AGES.

| | | | |
|---|---|---|---|
| Still-born, | 8 | Between 20 years and 30 years, | 27 |
| 1 day, | 2 | " 30 " " 40 " | 18 |
| 1 day and 30 days, | 5 | " 40 " " 50 " | 14 |
| Between 1 month and 6 months, | 21 | " 50 " " 60 " | 2 |
| " 6 months and 12 " | 13 | " 60 " " 70 " | 7 |
| " 1 year " 3 years, | 29 | " 70 " " 80 " | 1 |
| " 3 " " 5 " | 8 | " 80 " " 90 " | 0 |
| " 5 " " 10 " | 7 | " 90 " " 100 " | 0 |
| " 10 " " 20 " | 8 | " 100 " | 0 |
| Total number under 10 years, | 93 | Total number over 10 years, | 77 |

Ages not given, 2 Total, 170

### NATIVITIES.

| | | | |
|---|---|---|---|
| American, | 73 | Prussian, | 1 |
| German, | 43 | Welsh, | 0 |
| Irish, | 29 | French, | 2 |
| English, | 2 | Scotch, | 1 |
| Canadian, | 0 | Holland, | 1 |
| Swiss, | 1 | Country not given, | 17 |

Total, 170

